# Mining the Virgin Land of Neurotoxicology: A Novel Paradigm of Neurotoxic Peptides Action on Glycosylated Voltage-Gated Sodium Channels

**DOI:** 10.1155/2012/843787

**Published:** 2012-07-08

**Authors:** Zhirui Liu, Jie Tao, Pin Ye, Yonghua Ji

**Affiliations:** Laboratory of Neuropharmacology and Neurotoxicology, Shanghai University, Nanchen Road 333, Shanghai 200444, China

## Abstract

Voltage-gated sodium channels (VGSCs) are important membrane protein carrying on the molecular basis for action potentials (AP) in neuronal firings. Even though the structure-function studies were the most pursued spots, the posttranslation modification processes, such as glycosylation, phosphorylation, and alternative splicing associating with channel functions captured less eyesights. The accumulative research suggested an interaction between the sialic acids chains and ion-permeable pores, giving rise to subtle but significant impacts on channel gating. Sodium channel-specific neurotoxic toxins, a family of long-chain polypeptides originated from venomous animals, are found to potentially share the binding sites adjacent to glycosylated region on VGSCs. Thus, an interaction between toxin and glycosylated VGSC might hopefully join the campaign to approach the role of glycosylation in modulating VGSCs-involved neuronal network activity. This paper will cover the state-of-the-art advances of researches on glycosylation-mediated VGSCs function and the possible underlying mechanisms of interactions between toxin and glycosylated VGSCs, which may therefore, fulfill the knowledge in identifying the pharmacological targets and therapeutic values of VGSCs.

## 1. Introduction

**Figure 1:**
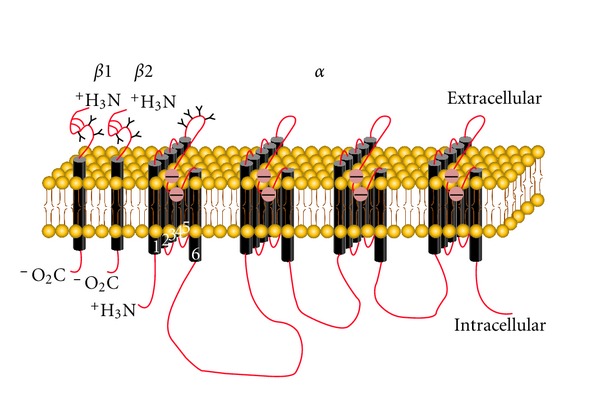
Structure and localization of glycosylation sites of VGSC. The primary structures of the subunits of the voltage-gated ion channels are illustrated as transmembrane-folding diagrams. Cylinders represent probable *α*-helical segments. Bold lines in red represent the polypeptide chains of each subunit, with length approximately proportional to the number of amino acid residues in the brain sodium channel subtypes. The extracellular domains of the *β*1 and *β*2 subunits are shown as immunoglobulin-like folds. Sites of probable N-linked glycosylation; open circle with “—”, amino residues that form the ion selectivity filter and tetrodotoxin binding site.

In neurons and most excitable cells, multiform action potentials driven by depolarizing neuronal firing are considered to be accounted by spatiotemporal activation and integral performances of tissue-specific VGSCs [1]. Generally, VGSCs consist of an *α* subunit (260 kDa) and several auxiliary *β* subunits (*β*1–*β*4, 33–38 kDa). The *α* subunit is well organized in four homologous domains (DI–DIV) which contain six transmembrane segments each (S1–S6). The hairpin-like loop between S5 and S6 segments region is functioned as ion-permeable pore [2]. Notably, both *α* and *β* subunits are highly glycosylated cross-membrane proteins [3–7] (Figure 1).

The most common form of glycosylation sites on VGSCs protein is mainly composed by N-linked sialic acids, [8]. Estimation indicates that 15%–40% of the total VGSC *α* subunit molecular weight is carbohydrate [6, 7, 9]. Approximately 40%–45% of the added carbohydrate residues are sialic acid moieties, resulting in the addition of an estimated 100 sialic acid residues per subunit molecule [6, 9]. Up to now, through bioinformatics prediction, there have been found tens of potential extracellular glycosylation sites located mainly within the pore region lining between DI S5-S6 in *α* subunit of VGSCs, less of which, however, have been funtionally characterized [5, 10, 11]. Far less than *α* subunit, only three of the four N-linked glycosylation sites present in the N terminus of *β* subunits are thought to be glycosylated in the mature protein [5, 12].

Glycosylation has long be known to participate in regulation of functional expression of channels, such as folding, trafficking, and membrane-insert localization, but also influence electrophysiological properties [13–16]. Inhibition of N-linked glycosylation altered the voltage dependence of channel gating of K_v_1.1 and K_v_LQT/minK (IsK) and the open probability of the renal outer medullary K^+^ channel ROMK1 (inward rectifier K^+^ channel), as well as the pH sensitivity of K_v_LQT/minK channels [17, 18]. In addition, N-linked glycosylation was found to be capable of increasing the stability of Shaker potassium channels proteins in trafficking from the endoplasmatic reticulum to the Golgi [13, 15]. Comparably, the information of glycosylation on modulating VGSCs function was less approached. The current knowledge about the modulation of glycosylation on VGSCs is mainly focused on reducing the voltage-dependent gating sensitivity, and thereby lowering the AP threshold at neuronal network level [19–21]. But questions like (1) how does glycosylation control the local static milieu on the surface of VGSCs protein which is attributed to the state of cell membrane? (2) what about the subtype-specific modulation of glycosylation on VGSCs? (3) does each glycosylation site modulate equally or synergically on the VGSCs gating? still appear as difficult tasks to work out.

To address the above problems, one may resort to segment-swap chimera construction or glycosylation-deficient cells to reduce the level of glycosylation [10, 22]. However, such methods may still bring about the unexpected artificial factors. More importantly, the complexity of extracellular environment and structure of channel protein itself may also disturb the reliability. Therefore, one prospective and efficient way is to find out the subtype-specific glycosylation modulators.

Natural toxic polypeptides originated from various venomous animals are deemed to be specifically targeting on VGSCs by either lowering the threshold for activation or delaying the inactivation process [23–26]. To date, there have been found six receptor sites of these toxins on VGSCs, some of which are even residing in the overlapped region of glycosylated sites on VGSCs [23, 26–28]. Meanwhile, the pharmacological studies have demonstrated that the binding of these toxins and their targets is highly subtype specific [26, 27, 29]. Thus, they are hopefully utilized as efficient tools to more precisely uncover the role of glycosylation on VGSCs gating and the overall performances on channel pathology in clinical therapy.

## 2. Mechanism of Glycosylation to the Voltage Dependence of VGSCs Gating

A general understanding about the physiological function of glycosylation on VGSCs is to control the voltage sensitivity in a channel through the number of sialic acids residing on [30]. Once these sialic acids are removed by deglycosyslated reagents, such as tunicamycin and neuraminidase, the voltage-dependent activation will shift to a more depolarized direction and thereby raising the threshold for AP generation [7, 22]. Currently, one commonly accepted notion is that it was negative charges brought about by a significant content of sialic acid residues on the glycosylation sites of the extracellular region that caused the hyperpolarized voltage dependence of gating [22]. However, some reports have suggested that various Na_v_ *α* subunits are differently glycosylated/sialylated even expressed in the same cell line [19]. This difference in *α* subunit sialylation directly and differently alters channel gating [10]. Here, two mechanisms describing the differential modulation of glycosylation on VGSCs gating are discussed below.

### 2.1. “Subtype-Specific” Mechanism

It is well known that VGSCs have nine tissue or developmentally distinct subtypes, named as Na_v_1.1 to Na_v_1.9, each of which has been found to be responsible for functionally diverse electric activities within the specific region they are expressed [31]. Recent researches have indicated that different VGSC subtypes have differential responses to the glycosylation.

Deglycosylated Na_v_1.4 could result in a depolarizing shift in both voltage-dependent activation and inactivation. Comparatively, deglycosylated Na_v_1.5 could lead to a depolarized voltage-dependent activation but not inactivation [20, 21]. By contrast, deglycosylation could only shift the midpoint of steady-state inactivation of Na_v_1.9 in adult small DRG neurons to more depolarized potentials [20]. Gating of Na_v_1.2 and Na_v_1.7 could not be significantly affected by deglycosylation [32]. Our recent work found that the steady-state activation curve of deglycosylated Na_v_1.3 was depolarized to a more positive direction, while inactivation curve was negatively shifted [33]. Thus far, glycosylation is capable of modulating VGSCs gating to various extents.

There was a report which attributed such differential modulation of glycosylation to distinct extent of glycosylation of each VGSC subtype. Immunoblot data suggested that Na_v_1.4 is more glycosylated than Na_v_1.5 [10]. As a matter of fact, Na_v_1.1–Na_v_1.4, are heavily glycosylated (about 15~30%), whilst Na_v_1.5 and Na_v_1.9 are barely glycosylated (~5%) [20]. Accordingly, it was indicated that Na_v_1.4 voltage-dependent gating parameters are significantly and essentially uniformly altered by sialic acid than that of Na_v_1.5. For the reason that the external surface of one VGSC *α*-subunit was estimated to have about 110–130 negative charges composed by the sialic acids (amount to about 40% of total carbohydrate in VGSC) [32], it is likely that sialic acid alters the electric field sensed by the gating mechanism of the channel [10]. That is, a higher level of glycosylation in VGSC may lead to more depolarized shift in voltage-dependent gating when deglycosylated. As a consequence, the deglycosylated VGSCs may require larger depolarizing stimulus to activate.

### 2.2. “Cell-Specific” Mechanism

One seemly contradictory notion against the “subtype-specific” mechanism was that it was because of certain internal environment of different cells types which differentially produce sialylated proteins leading to a spectrum of Na_v_ functional sialic acid levels that directly modulate channel gating. Hence, such mechanism was termed as “cell specific” [19, 32].

 “Cell-specific” may arise from two compensated factors. One is that the glycosylation extent in certain VGSC protein may vary throughout the developmental stages: for example, Na^+^ currents in adult rat cortical and dorsal root ganglion neurons are less sensitive to sialic acid than Na^+^ currents from neonatal neurons [20, 34]. Adult ventricular myocyte VGSCs were more heavily sialylated and gated at more hyperpolarized potentials than they were in neonate ventricular myocyte VGSCs. One possible explanation for this increased sialylation would be a chronic increase in sialyltransferase activity in the developing ventricles [19].

Another aspect resulting in differential glycosylation came from the tissue- or cell-type-specific proteins that modify the number of sialic acids on the surface of VGSCs. One recent work have suggested that the modulation of glycosylation may display a rather complex profile with the combinatorial link with VGSC *β*-subunit: for example, when *β*1 subunit was coexpressed with Na_v_1.2, Na_v_1.4, Na_v_1.5, and Na_v_1.7, the extent of hyperpolarized shift in voltage-dependent activation has the following order: Na_v_1.7 > Na_v_1.5 > Na_v_1.2 > Na_v_1.4, where Na_v_1.4 was less modulated by *β*1 subunit, which indicated an essentially saturating level of functional sialic acids. On the contrary, Na_v_1.7 alone contain the least functional sialic acids in DIS5-S6, and therefore the levels of functional sialic acids increase, causing the most remarkably hyperpolarization in voltage-dependent activation when coexpressed with *β*1 subunit [32].

## 3. Novel Paradigm of Interaction between VGSCs and Neurotoxic Peptides

Since the distinct binding affinity with VGSCs, natural toxins such as that from marine animals (saxitoxin, sea anemone) and arthropods (scorpion toxins, spider toxins) have long been applied to investigate the structure-function relationship of VGSCs and regarded as pharmacological templates for developing therapeutic leads [23–26, 35].

However, there have been paradoxes seen in contact cells and in vitro studies on VGSCs. For instance, BmK I, a site-3-specific modulator of VGSCs from scorpion *Buthus martensii* Karsch (BmK) was capable to prevent the inactivation of Na_v_1.2 and produce persistent current, which may account for BmK I-induced epileptiform responses in rats [36–39]. However, the specific binding of BmK I to the VGSCs in rat brain synaptosomes (mainly Na_v_1.2) was undetectable [40]. Although the contradictory modulation of BmK I between VGSCs-rich synaptosomes and heterologously expressed Na_v_1.2 may somewhat attribute to lack of BmK I-sensitive VGSCs [37], the complex intracellular enzymatic context of synaptosomes stimulated us to speculate the involvement of glycosylation on modulating sensitivity of VGSCs to BmK I.

**Figure 2:**
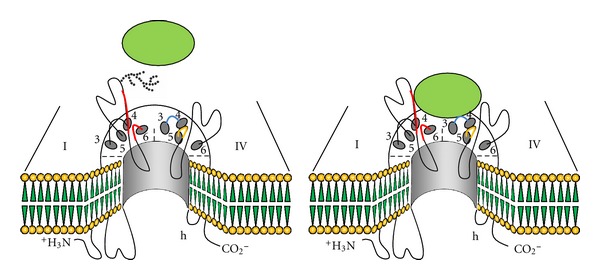
Hypothesis on deglycosylation-created modulation of scorpion toxins to VGSCs. For glycosylated VGSC, the sialic carbohydrates (dotted branch) shield the binding site and thus prevent toxin binding (left). If the sialic carbohydrates are deleted, the toxin accessibility to the binding site is facilitated because of the absence of the oligosaccharide groups and consequently modulate the gating of VGSCs (right).

In our recent researches, it was found that BmK I could distinctively modulate glycosylated/deglycosylated Na_v_1.2 expressed in oocytes. The voltage dependent activation of deglycosylated Na_v_1.2 was significantly shifted to more negative direction by BmK I, contrary to the little effects on the glycosylated ones. This research indicated that the glycosylated sites on VGSCs may act as umbrellas to shield the interaction sites of VGSCs with BmK I, and if biochemically removed, may facilitate the binding of BmK I to the receptor sites (Figure 2).

Coincidentally, as a receptor site-3-specific modulator, BmK I has been suggested to be capable of binding to the region where glycosylation sites also reside [37, 40]. It has been indicated that the extracellular loops between transmembrane segments S5 and S6 in domain I of the *α*-subunit are involved in the formation of receptor site 3 [28]. In addition, several antibodies that recognize the extracellular loops between transmembrane S5 and S6 in domains I and IV prevent *α*-scorpion toxin binding to receptor site 3, suggesting involvement of amino acid residues in these locations [41]. All these clues may potentially support the notion that there exists interaction between glycosylation sites and BmK I, by which the binding affinities of BmK I to VGSCs may be altered if the channel was decosylated.

Similar phenomenon can also be referred to the mode of actions of sulfamethoxazole (SMX) on HERG channel accompanying with a mutant subunit MiRP1 (T8A). Under normal conditions, the carbohydrates were attached to shield the variable receptor and thus impairing the SMX binding. Conversely, in channels formed with MiRP1 T8A mutants that are deficient in glycosylated sites by mutation, SMX accessibility to the receptor is facilitated by the absence of the oligosaccharide groups [42].

Likewise, Catterall et al. in 1987 have concluded the possible mechanism to explain the lack of binding of saxitoxin, one VGSCs-specific blocker, to the glycosylated VGSCs: the negative surface charges on the glycosylated sodium channel are expected to increase the local concentration of Na^+^ near the extracellular opening of its transmembrane pore and to increase the local concentration of a cationic ligand like saxitoxin near its receptor site. The lack of effect of inhibition of sialylation with castanospermine on saxitoxin binding indicates that the saxitoxin receptor site is located distance from the negative surface charges contributed by sialic acid residues or is insulated from their effects by the protein structure [7].

## 4. Perspectives

Several lines of evidence suggest that regulation of the level of sialylation is a powerful mechanism to control the surface charge of channels as well as neuronal channel pathology [27, 43–45]: for example, long QT syndrome (LQT, a cardiac disease relate to HERG) is also caused by the mutation of glycosylated sites in HERG [45]. It was reported that glycosylation is essential for the biosynthesis and the maintenance of functional VGSCs in fibroblastoma cells [46]. Additionally, one of main pathogenesis resulting in sinus arrhythmia is insufficient level of glycosylation in cardiac VGSCs [47]. Moreover, some studies showed that the chronic pain is related to the increase of glycosylation in neuron membrane in nerve injury sites [48]. Finally, an increase in sialic acid-negative surface charge resulting in reduced AP threshold and increased excitability could be one of the important factors of the pathogenesis of epilepsy associated with these inherited disorders [22].

The researches on detecting the modulation of glycosylation on VGSCs function are subjected to two major challenges. (1) There still lack of specific tools to precisely and efficiently modify the level of glycosylation on VGSCs, which may result in ambiguous observations in the real studies. Maybe some bioinformatics predictions could provide the auxiliary clues to compensate this problem. However, it remains to be more cautious in treating the computer-assisted data even until some site-directed evidence has been conducted. (2) The static alteration in glycosylation sites can restructure the receptor sites in VGSCs where some potential specific drugs may bind with. Thus, it is a critical task to discriminate the possible disturbance of glycosylation when treating with some seemly inefficient drugs targeting on VGSCs.

As sodium channel-specific modulators and neurotoxins have great potentials to probe the intriguing subtle structural variations in VGSCs found across different tissues and species. However, the pharmacological sensitivity of VGSCs toward toxins, seen in Section 3, may be subject to differential modulation due to the glycosylation of VGSC protein itself, leading to the difficulties in observing the actual interactions. Hence, a broader view of how neurotoxins modulate neuronal activities and thereby valuable information regarding to VGSCs as therapeutic targets could be obtained until more precise details about the role of glycosylation in determining toxin-channel interaction be deduced.
